# LRG-1 promotes pancreatic cancer growth and metastasis via modulation of the EGFR/p38 signaling

**DOI:** 10.1186/s13046-019-1088-0

**Published:** 2019-02-13

**Authors:** Zhi-Bo Xie, Yi-Fan Zhang, Chen Jin, Yi-Shen Mao, De-Liang Fu

**Affiliations:** 1 Department of Pancreatic Surgery, Huashan Hospital, Fudan University, 12 Central Urumqi Road, Shanghai, 200040 China; 2 Department of Plastic & Reconstructive Surgery, Shanghai Ninth People’s Hospital, School of Medicine, Shanghai Jiao Tong University, 639 ZhizaojuRoad, Shanghai, 200011 China

**Keywords:** LRG-1, Pancreatic ductal adenocarcinoma, Epidermal growth factor receptor (EGFR), p38, Biomarker

## Abstract

**Background:**

The abnormal expression of leucine-rich-alpha-2-glycoprotein 1 (LRG-1) is reported to be associated with multiple malignancies, but its role in the progression of pancreatic ductal adenocarcinoma (PDAC) remains to be determined.

**Methods:**

The expression of LRG-1 was assessed in PDAC tissues by RT-PCR, Western blot and immunohistochemistry. LRG-1-silenced or overexpressed cell lines were constructed using shRNA or LRG-1-overexpressing plasmids. EdU incorporation assay, Transwell invasion and wound-healing assays were performed to evaluate the proliferation, invasion and migration of PDAC cells. In addition, protein expression of the mitogen-activated protein kinase (MAPK) pathway was detected using Western blot. Finally, Co-immunoprecipitation assay was conducted in search of the potential interaction between LRG-1 and epidermal growth factor receptor (EGFR).

**Results:**

The expression of LRG-1 in PDAC tissue was significantly higher than that in adjacent normal tissue, and high LRG-1 expression predicted poor survival and a late tumor stage. In addition, LRG-1 markedly promoted the viability, proliferation, migration and invasion of PDAC cells in vitro and facilitated tumor growth in vivo. More importantly, we revealed that these bioactivities of LRG-1 might result from its selective interaction with EGFR, which might further activate the p38/MAPK signaling pathways.

**Conclusion:**

LRG-1 may prove to be a promising biomarker for predicting prognosis of PDAC patients. Inhibition of LRG-1 or its downstream pathway could be a potential therapeutic target for the treatment of PDAC.

## Introduction

Pancreatic ductal adenocarcinoma (PDAC) is one of the most lethal malignancies [1] with a median survival less than 6 months and a 5-year survival rate of less than 6% [2–5]. The incidence of PDAC is increasing yearly. According to GLOBOCAN estimates, there were 458,918 newly diagnosed cases of pancreatic cancer globally in 2018 [6]. The dismal prognosis of PDAC has persisted for decades, with only minimal improvement in these years, which might be ascribed to the ambiguous mechanism underlying the development and progression of PDAC [7].

A variety of signaling pathways have been reported to be involved in the development and progression of PDAC including mitogen-activated protein kinase (MAPK) [8] and Notch [9] signaling pathways, growth factors such as epidermal growth factor (EGF) [8], fibroblast growth factor (FGF) [10] and insulin-like growth factor 1 (IGF-1) [11]. Based on these findings, scientists have uncovered various biological targets, and developed several corresponding targeted therapies [12, 13]. However, concerns regarding their low efficacy, adverse events and high risk of recurrence are frustrating challenges for clinicians. This deficiency is primarily due to the poor understanding about the exact pathophysiology and the key driven gene in the initiation and development of PDAC.

In 1977, Leucine-rich-alpha-2-glycoprotein-1 (LRG-1) was first identified as an inflammatory protein in human serum by Haupt and Baudner [14]. However, few related studies have been reported and the function of LRG-1 remained unknown until 2013, when Wang et al. reported that LRG-1 was able to promote angiogenesis by modulating endothelial transforming growth factor β (TGF-β) signaling [15]. Since then, more studies on LRG-1 have emerged gradually and LRG-1 is recognized as a new regulator of tumorigenesis and a novel oncogene-associated protein [15, 16], playing an important role in epithelial-mesenchymal transition (EMT) and angiogenesis in colon cancer [16, 17]. LRG-1 is also known as a promising tumor biomarker and an independent prognostic factor for endometrial carcinoma [18] and non-small cell lung cancer [19]. In addition, studies demonstrated that LRG-1 promoted glioma cell invasion, migration and angiogenesis in the damaged retina [20]. Some recent studies demonstrated that serum LRG-1 level was significantly increased in PDAC patients and was correlated with progressive clinical stages [21]. Another study reported that the combination of tissue inhibitors of metalloproteinase-1 (TIMP-1), and LRG-1 to carbohydrate antigen 19–9 (CA 19–9) statistically significantly improves the detection of early-stage PDAC [22]. However, the prognostic value of LRG-1 in PDAC patients has not been reported and the effect of LRG-1 on human PDAC cells and its potential molecular mechanisms remain largely unknown.

The aim of the present study was to examine LRG-1 expression in tissue samples, evaluate the prognostic value of LRG-1 in PDAC patients, clarify the effect of LRG-1 on various cellular behaviors of PDAC cell lines both in vivo and in vitro, and explore potential signaling pathways and target proteins involved in the biochemical mechanism underlying the regulatory effect of LRG-1 on the pathogenesis of PDAC.

## Materials and methods

### Patients

This retrospective study involved 127 consecutive PDAC patients admitted to our hospital between 2011 and 2013. Patients enrolled in this study should be (a) aged 18–75 years; (b) without distant metastasis; (c) diagnosed with resectable PDAC and confirmed by postoperative pathology. Patients with the following criteria were excluded: (a) a previous history of treatment; (b) multi-organ dysfunction; and (c) contradictions for pancreatic surgery. All patients underwent a baseline assessment within one week before pancreatic surgery.

### Cell culture

Human PDAC cell lines BxPc-3 and SW1990 were purchased from the ATCC (American Type Culture Collection). Cells were cultured in DMEM (Gibco, Grand Island, New York, USA) supplemented with 10% fetal bovine serum (FBS, Gibco), 1% penicillin and 1% streptomycin, and incubated at 37 °C in a humidified atmosphere with 5% CO_2_.

### RNA extraction and qRT-PCR

Total RNA was isolated from the PDAC and adjacent normal specimens using TRIzol reagent (Invitrogen, Carlsbad, CA, USA). The RNA was reversely transcribed into cDNA with Oligo (dT) and M-MLV Reverse Transcriptase (Thermo Fisher Scientific). GAPDH was used as a reference gene. Primers of LRG-1 were as follows: 5’-GGACACCCTGGTATTGAAAGAAA-3′ (forward) and 5’-TAGCCGTTCTAATTGCAGCGG-3′ (reverse) [23].

### Immunohistochemistry and evaluation

Paraformaldehyde-fixed paraffin-embedded tissue sections (5 μm) were prepared using a rabbit monoclonal immunoglobulin IgG specific for LRG-1 or EGFR or p-p38 (Abcam, Cambridge, UK, 1:200) and incubated overnight at 4 °C. The sections were developed with diaminobenzidine and counterstained with haematoxylin after incubation with secondary antibodies [24].

### Western blot

Tissues lysates were electrophoresed on SDS-PAGE gel and transferred to PVDF membranes (Millipore, Bedford, MA, USA). The membranes were blocked with 5% skim milk and the primary antibodies were used for incubation overnight at 4 °C. After washing, PVDF membranes were incubated with secondary antibodies for 1 h. Immunoreactive bands were quantitatively analyzed with ImageJ software (http://imagej.nih.gov/ij/) [24]. The primary antibodies were as follows: anti-LRG-1, 1:1000; anti-Smad1/5, 1:1000 (Abcam, Cambridge, UK); anti-GAPDH, 1:10000; anti-MMP-2, 1:1000; anti-MMP-9, 1:1000; anti-TIMP-1, 1:1000; anti-JNK, 1:1000; anti-p-JNK, 1:1000; anti-ERK, 1:1000; anti-p-ERK, 1:1000; anti-p38, 1:1000; anti-p-p38, 1:1000; anti-p-Smad1/5, 1:1000 (Cell Signaling Technology, Beverly, MA, USA).

### LRG-1 knockdown and over-expressing in PDAC cells

The LRG-1 shRNA was constructed in the pLKO.1 shRNA expression vector. Purified plasmids were transfected into HEK-293 T cells by using Lipofectamine 3000 (Invitrogen, Carlsbad, CA, USA), along with helper plasmids psPAX2 and VSV-G. The virus supernatant was added on the cell culture in the presence of 8 mg/ml polybrene. Positive clones were obtained upon puromycin selection. The interference sequence of shRNA was as follows: 5’-AGCTAAAAAGATGTTTTCCCAGAATGACTCTCTTGA AGTCATTCTGGGAAAACATCGGG-3′ (shRNA-1), 5’-GATCCCCGATGTTTTCCCAGAATGACTTCAAGAGAGTCATTCTGGGAAAACATCTTTTT-3′ (shRNA-2).

The amplified LRG-1 coding region was cloned into the pUM-T vector, and positive recombinant plasmid was sequenced. Cells were transfected and selected for stable expression clones as described above. The amplification primers for the LRG-1 coding region were as follows: forward: 5’-GGCTGAAGCTTGCAGAGCTACCATGTCCTC-3′; reverse: 5’-TGATGGATCCTGGTCTCACTGGGACTTGG-3′.

### Thiazolyl blue tetrazolium bromide (MTT) assay

For the cell viability assay, cells were seeded in 96-well plates (2.5 × 10^3^ cells per well). After 24, 48, or 72 h, cell culture medium was replaced by MTT working solution, followed by 4 h incubation at 37 °C in a 5% CO_2_ incubator (Sigma-Aldrich Corp., St. Louis, MO, USA). After removing the MTT working solution and adding the DMSO, the absorbance at 490 nm were detected using a microplate reader (Tecan Group AG, Männedorf, Switzerland).

### EdU incorporation assay

Click-iT EdU (5-ethynyl-2′-deoxyuridine) Alexa Fluor 488 Imaging Kit (Invitrogen, Carlsbad, CA, USA) was used to detect PDAC cell line proliferation according to the manufacturer’s instructions. Fluorescence was analyzed using a Zeiss 510 laser-scanning microscope (Zeiss, Thornwood, NY, USA) [24].

### Wound-healing assay

PDAC cell lines were seeded in six-well plates (5 × 10^5^ cells per well). A scratch wound was created using a micropipette tip when cells reached confluence. The narrowing of the wound area was investigated at 0 and 48 h. The area of each wound was measured via ImageJ software.

### Transwell assays

The 48-well Transwell plates (Millipore, Bedford, MA, USA) with 8 μm pore filters were used for measuring cell migration. A total of 1 × 10^6^/well PDAC cells were seeded in the upper chambers and then incubated with medium alone at 37 °C in a 5% CO_2_-filled incubator. PDAC cells migrating to the lower surface were stained with 0.5% crystal violet (Sigma-Aldrich, St. Louis, MO, USA) and photographed.

### Co-immunoprecipitation assays

Cell extracts was diluted in IP lysis buffer and incubated with 1.5 μg normal rabbit IgG for 2 h, followed by 2 h of incubation with 5 μl protein A magnetic beads (Millipore, Bedford, MA, USA) to precipitate proteins that interacted non-specifically with IgG and/or protein A magnetic beads. This pre-cleared lysate was then incubated with 2 μg anti-LRG-1 antibody (Abcam, Cambridge, UK), at room temperature overnight. Protein A magnetic bead (20 μl) was added and incubated at room temperature for 6 h. Beads were finally washed 3 times using lysis buffer and eluted by incubating the beads 5 min at 70 °C in 25 μl in NuPAGE LDS sample buffer. Immunoprecipitated proteins were analyzed by Western blot analysis.

### Xenograft mouse model

Totally 32 BALB/c nude mice (4–5 weeks old, 18–20 g) used in this study were obtained from Shanghai Medical College of Fudan University. All nude mice were routinely bred in a specific pathogen-free (SPF) laboratory in the animal center of Shanghai Medical College of Fudan University. The Mice were randomly divided into 4 groups: normal group, LRG-1-overexpressing group, LRG-1 shRNA-1 group and LRG-1 shRNA-2 group. The subcutaneous tumor models were established by respectively injecting 1 × 10^7^ cells suspension into the right upper flanks of BALB/c nude mice respectively. The tumor volume was calculated using the formula (width^2^ × length)/2. After four weeks, the mice were euthanized, and subcutaneous tumors were removed and fixed in 4% paraformaldehyde.

### Statistical analysis

SPSS 21.0 (IBM, Chicago, USA) was used to perform statistical analysis, and *P* < 0.05 was defined as the threshold of statistical significance. Normally distributed data were expressed as mean ± standard deviation (SD), and asymmetrically distributed data were expressed as median (range). Differences in outcomes between high and low expressions of LRG-1 were assessed for significance using independent-samples t tests. ROC curve was used to determine the sensitivity and specificity of prediction of LRG-1 for PDAC diagnosis. Kaplan-Meier method was used to calculate survival curves, and the significance was analyzed by log-rank test. Multivariate survival analysis was performed by Cox proportional hazards model.

## Results

### High LRG-1 expression predicts poor survival and late tumor stage

**Fig. 1:**
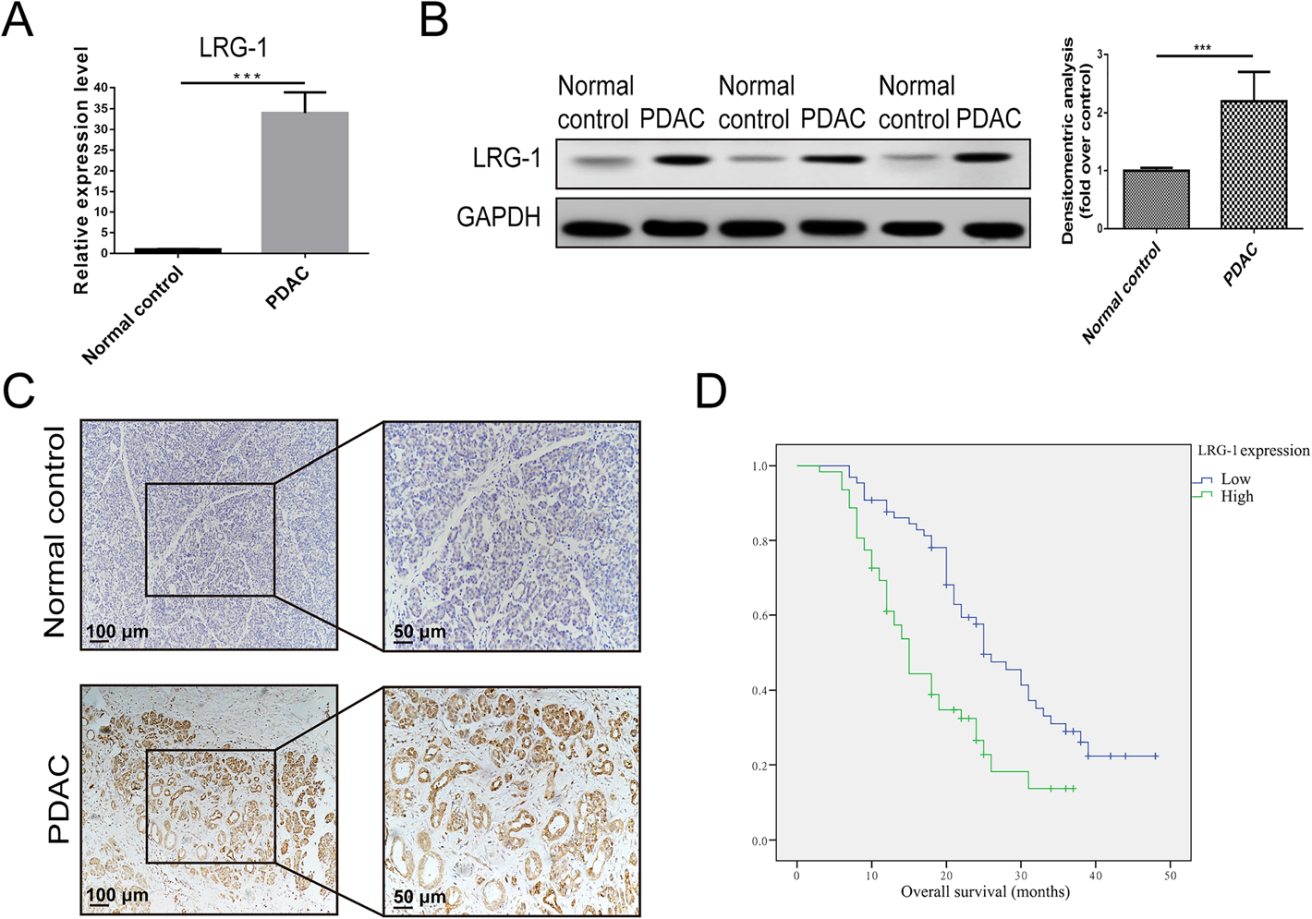
LRG-1 is overexpressed in PDAC tissues. Overexpression of LRG-1 (**a**) mRNA and (**b**) protein in PDAC tissues than normal tissues. **c** Representative images of immunohistochemical staining of LRG-1 in normal tissues and PDAC tissues. **d** Survival analysis of PDAC patients with different LRG-1 expressions. Data are presented as the mean ± SD; ***p < 0.001

The expression level of LRG-1 was first detected in PDAC and adjacent normal tissues. It was found that mRNA and protein expression of LRG-1 in PDAC tissue was significantly higher than that in the adjacent normal tissue (Fig. 1a, b). To obtain pathological evidence, immunohistochemistry (IHC) was subsequently performed, and the results showed that the expression of LRG-1 in PDAC tissue was higher than that in the adjacent normal tissue (Fig. 1c).

We further analyzed the expression of LRG-1 by IHC and other clinical factors in 127 PDAC patients. Our data showed that high LRG-1 expression (*P* = 0.001), late tumor stage (*P* < 0.001), and CA 19–9 levels (*P* = 0.020) were significant independent prognostic factors (Table 1). Patients with high LRG-1 levels had significantly poor survival than those with low LRG-1 levels (mean survival: 18.13 months vs. 28.66 months, *P* < 0.001) (Fig. 1d, Table 1). In addition, the logistic regression model showed that a higher LRG-1 expression was correlated with a later tumor stage (HR = 1.95, *P* = 0.034), a higher CA 125 (HR = 2.92, *P* = 0.023) and CA 19–9 level (HR = 1.70, *P* = 0.028) (Table 2).

**Table 1:** Prognostic value of different risk factors

Risk factors	Univariate regression analysis			Multivariate regression analysis			Overall survival	
	HR	95% CI	*P* value	HR	95% CI	*P* value	Mean ± SD, months	*P* value
LRG-1 expression								
Group 1: Low	2.29	1.47–3.57	<0.001	2.30	1.43–3.71	0.001	28.66 ± 1.73	<0.001
Group 2: High							18.13 ± 1.40	
Tumor stage								
Group 1: Stage I	3.02	1.97–4.62	<0.001	2.48	1.59–3.87	<0.001	32.08 ± 2.53	<0.001
Group 2: Stage II							22.41 ± 1.37	
Group 3: Stage III							10.08 ± 1.24	
R1 resection								
Group 1: Yes	2.87	1.23–6.66	0.015	2.16	0.89–5.25	0.089	12.50 ± 2.48	0.009
Group 2: No							24.88 ± 1.36	
Carbohydrate antigen 125								
Group 1: <35	2.04	1.25–3.32	0.004	1.39	0.82–2.34	0.219	26.06 ± 1.51	0.003
Group 2: ≥35							16.91 ± 2.06	
Carbohydrate antigen 19–9								
Group 1: <37	1.68	1.26–2.24	<0.001	1.42	1.06–1.90	0.020	31.01 ± 2.67	0.001
Group 2: 37–200							23.78 ± 1.55	
Group 3: >200							18.00 ± 1.89

**Table 2:** Risk factors correlated with LRG-1 expression using Logistic regression model

Risk factors	HR	95% Confidence Interval	*P* value
Tumor stage	1.95	1.05–3.61	0.034
R1 resection	8.15	0.97–68.27	0.053
Carbohydrate antigen 125	2.92	1.16–7.32	0.023
Carbohydrate antigen 19–9	1.70	1.06–2.72	0.028

### LRG-1 promotes PDAC cell viability, proliferation, invasion and migration

**Fig. 2:**
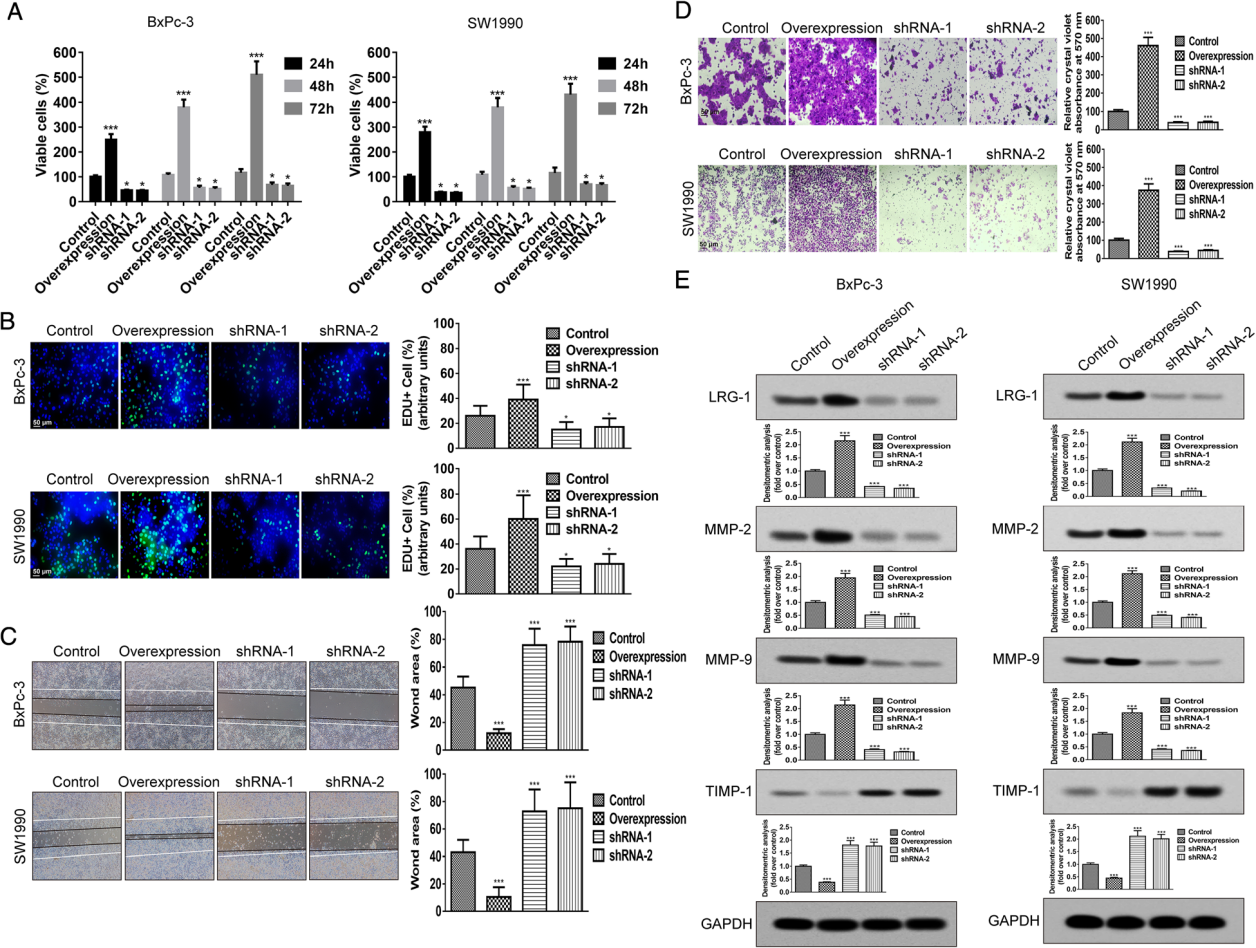
LRG-1 promotes PDAC cell viability, proliferation, invasion and migration **in vitro. a** MTT assays at 24, 48, or 72 h in LRG-1 knockdown and overexpression PDAC cells. **b** EdU incorporation assay at 48 h in LRG-1 knockdown and overexpression PDAC cells. EdU (green) was used to label proliferating cells and the nucleus was stained with DAPI (blue). Wound-healing assay (**c**) and Transwell cell invasion assay (**d**) at 48 h in LRG-1 knockdown and overexpression PDAC cells. **e** The protein level of MMP-2, MMP-9 and TIMP-1 at day 3 in LRG-1 knockdown and overexpression PDAC cells. Data are presented as the mean ± SD, *p < 0.05; **p < 0.01; ***p < 0.001

To determine the effect of LRG-1 in PDAC cells, LRG-1 knockdown or overexpressing experiments were performed in BxPc-3 and SW1990 cells. Once LRG-1 was attenuated by specific shRNA, the viability of BxPc-3 and SW1990 cells was significantly inhibited (Fig. 2a). Additionally, our EdU incorporation assay demonstrated that the proportion of EdU-positive cells decreased markedly after LRG-1 downregulation (Fig. 2b). Further wound healing assays revealed a profound migratory defect in LRG-1-depleted cells (Fig. 2c) and transwell assays observed a consistent reduction (Fig. 2d). Moreover, the downregulation of LRG-1 also reduced the expression of critical factors participating in tumor metastasis, including MMP-2 and MMP-9 (Fig. 2e). The expression of TIMP-1 was correspondingly up-regulated (Fig. 2E). In contrast, over-expression of LRG-1 could significantly promote cell viability (Fig. 2a), proliferation (Fig. 2b), migration (Fig. 2c) and invasion (Fig. 2d). In addition, enhanced LRG-1 expressions also upregulated the expression of MMP-2 and MMP-9, and reduced TIMP-1 expression simultaneously (Fig. 2e).

### LRG-1 promotes PDAC growth in vivo

**Fig. 3:**
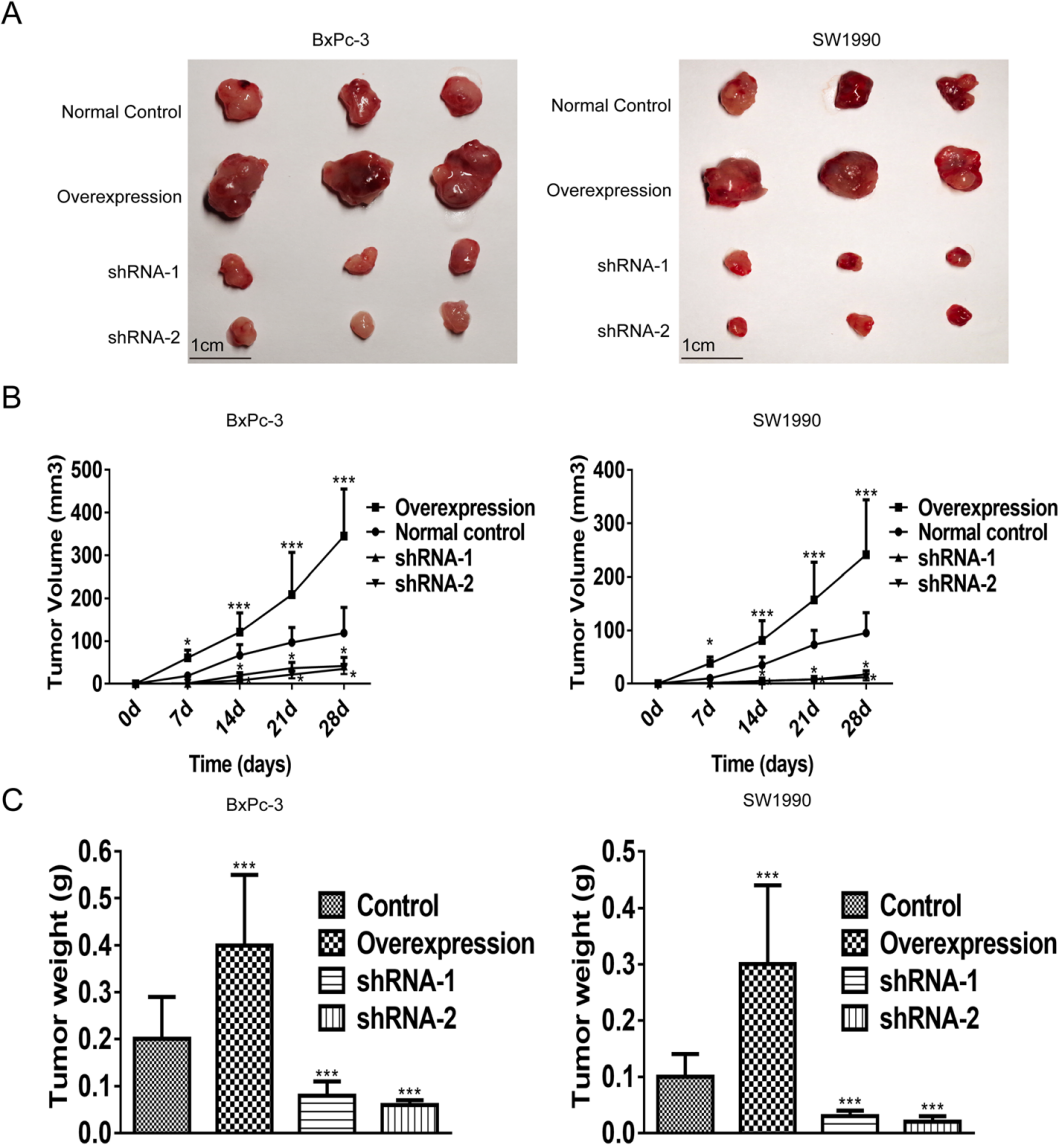
LRG-1 promotes PDAC growth in vivo. **a** Images of tumors at day 28 of the xenograft mouse model. **b** Tumor volume was measured using a caliper at indicated time points. **c** The tumor weight was measured at the end of the experiments. Data are presented as the mean ± SD, *p < 0.05; **p < 0.01; ***p < 0.001

To assess the function of LRG-1 in promoting the development of PDAC in vivo, a nude mice xenograft model was established and the results showed that both the tumor weight and size were reduced significantly at all designated time points in the nude mice injected with LRG-1 knock-down cells as compared with the nude mice injected with scramble plasmids (Fig. 3a, b, c). On the other hand, xenograft assays showed that the tumor burden in the nude mice injected with LRG-1 over-expressing PDAC cells was heavier than that in the nude mice injected with control cells (Fig. 3a, b, c). These data suggested that LRG-1 played an important role in the development and progression of PDAC in vivo.

### LRG-1 promotes PDAC cell proliferation and migration via p38/MAPK signaling pathways

**Fig. 4:**
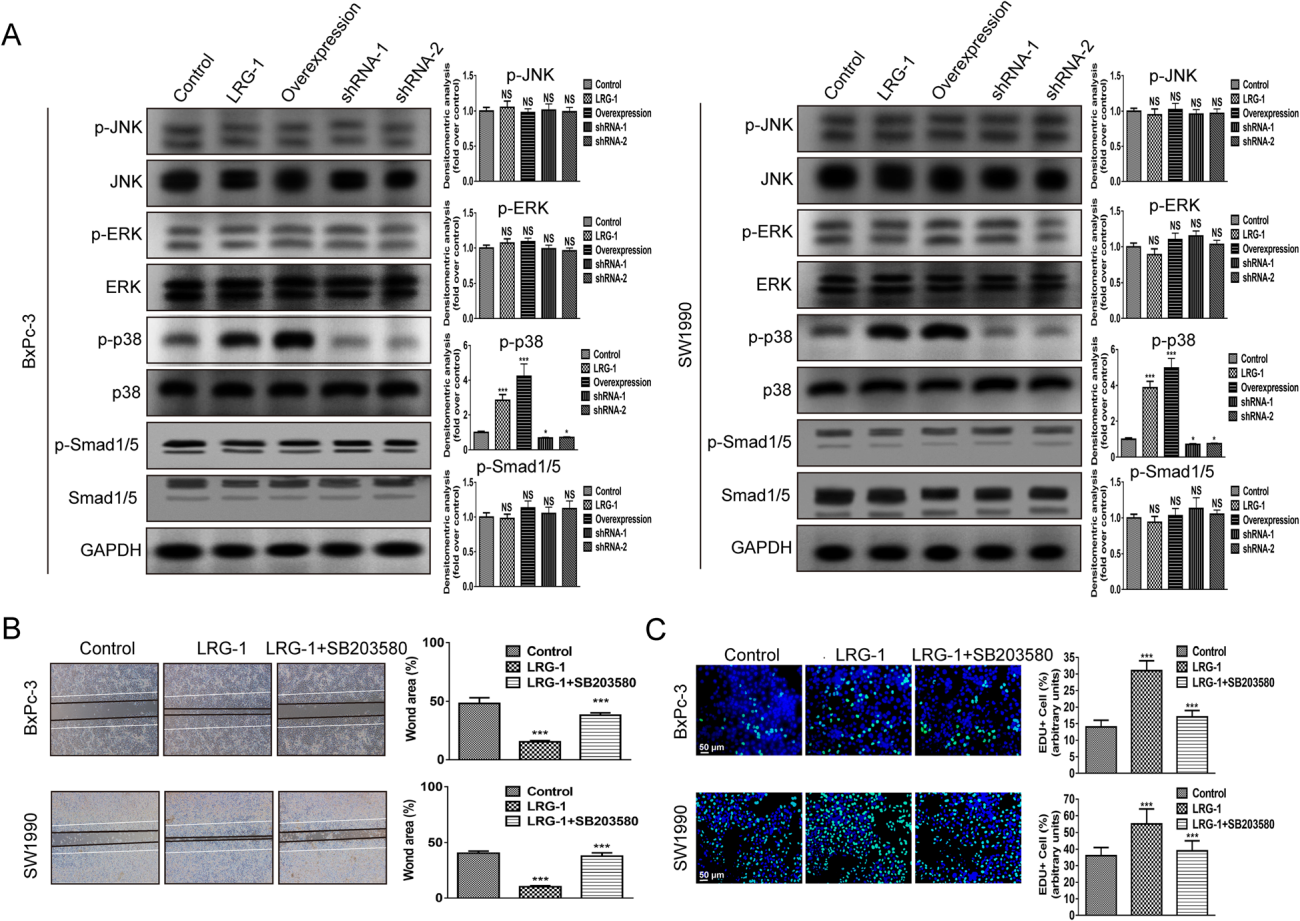
LRG-1 promotes PDAC cells proliferation and migration via p38/MAPK signaling. **a** Levels of phosphorylated and total JNK, ERK, p38 and Smad 1/5 were examined by Western blot after 30-min incubation of BxPc-3 and SW1990 cells with LRG-1 (500 ng/ml) for. Wound-healing assay (**b**) and EdU incorporation assay (**c**) in cultured PDAC cells after 48 h incubation with SB203580 (0.5 μM) and LRG-1 (500 ng/ml). Data are presented as the mean ± SD, NS = not significant; ***p < 0.001

MAPK pathway is known as one of the key signaling pathways that regulate the pathological process of pancreatic cancer [25, 26], and LRG-1 was reported to modulate the activation of MAPK signaling downstream protein [27]. To further explore the underlying mechanism of how LRG-1 promoted the malignant behaviors of PDAC cells, we analyzed the activation of the downstream mediators of the MAPK signaling pathway using Western blot assay, and found that LRG-1 incubation and LRG-1 overexpression significantly upregulated p38 phosphorylation in PDAC cells. The phosphorylation of p38 was remarkably decreased in LRG-1 knockdown PDAC cells (Fig. 4a). However, JNK and ERK phosphorylation were not obviously affected by LRG-1 (Fig. 4a). Previous study has reported that the addition of LRG-1 could induce Smad1/5 phosphorylation in endothelial cells in the presence of TGF-β1. Our Western blot assay demonstrated that exogenous LRG-1 could not significantly upregulate the expression level of p-Smad1/5 in human PDAC cells in the presence of TGF-β1 suggesting that LRG-1 activation in Smads signaling was not available in PDAC cells (Fig. 4a).

To confirm whether the enhancement of the malignant behavior of PDAC cells by LRG-1 was dependent on the activation of p38/MAPK signaling pathway, SB203580 (a specific inhibitor of this pathway) was used for the subsequent experiment. It was found that the promoting effect of LRG-1 on cell proliferation and migration was blocked after treatment of PDAC cells with LRG-1 and SB203580 (Fig. 4b, c) indicating that LRG-1 might promote the malignant behavior of PDAC cells via the p38/MAPK signaling pathway.

### EGF receptor (EGFR) mediates LRG-1 induced phosphorylation of p38 signaling

**Fig. 5:**
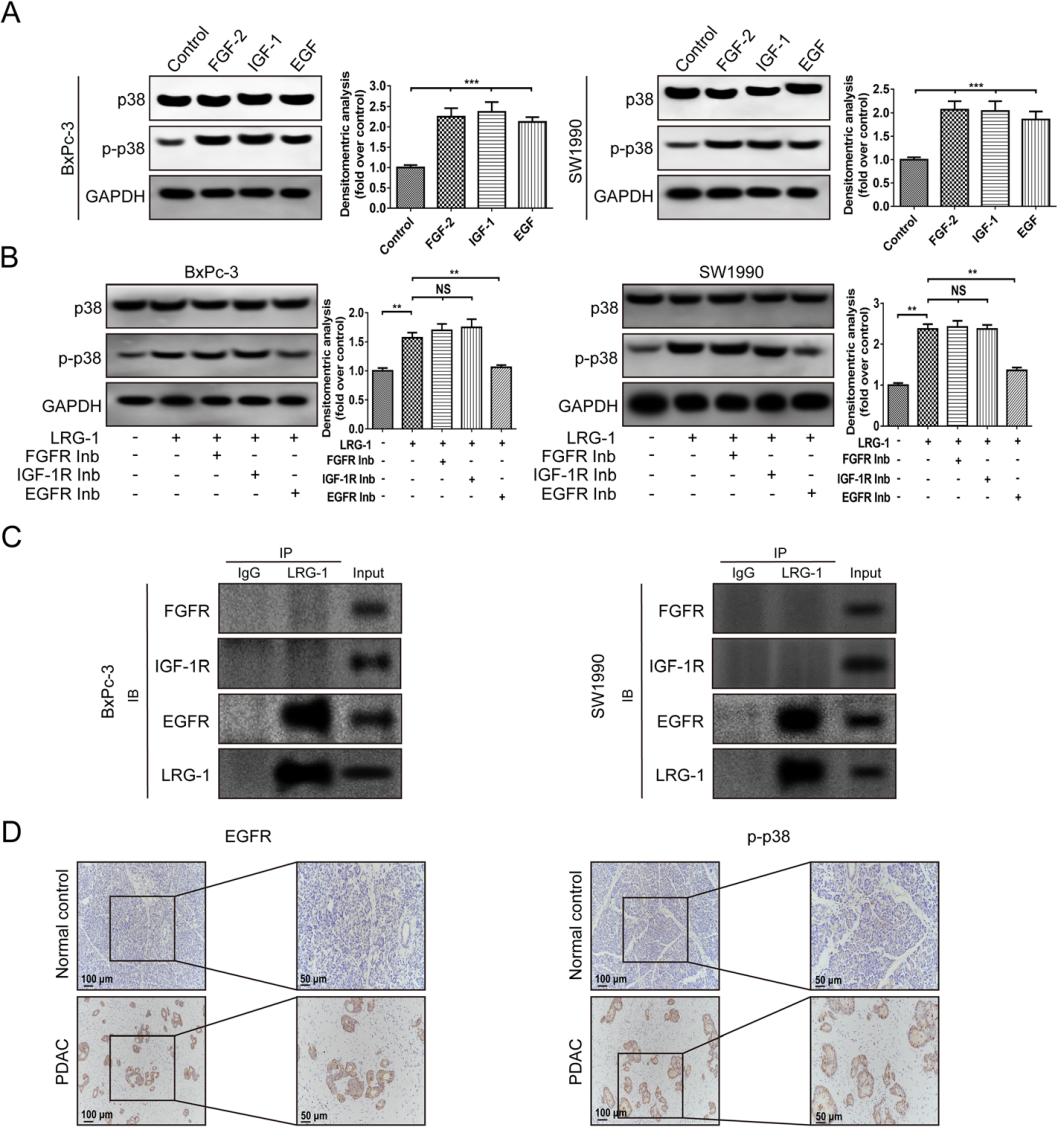
The role of EGFR in LRG-1-induced p38 phosphorylation. **a** Levels of phosphorylated and total p38 were examined by Western blot after 30-min incubation of cells with FGF-2 (20 ng/ml) or IGF-1 (100 ng/ml) or EGF (100 ng/ml). **b** The expression of phosphorylated and total p38 in both cell lines after 30-min incubation with LRG-1 (500 ng/ml) and FGFR inhibitor (PD173074, 0.2 μM) or IGF-1R inhibitor (OSI-906, 5 μM) or EGFR inhibitor (erlotinib, 5 μM). **c** Co-immunoprecipitation (Co-IP) between LRG-1 and FGFR or IGF-1R or EGFR. **d** Immunohistochemistry images of EGFR and p-p38 in human PDAC and normal pancreas tissues. Data are presented as the mean ± SD, NS = not significant; **p < 0.01; ***p < 0.001

During the pathological development and progression of PDAC, many growth factors play an important role, and the exaggerated activation of their downstream signaling has been found to be associated with the malignant behavior of PDAC cells [8, 28–31]. Among them, FGF [32], IGF [33] and EGF [8, 34] were the most important cytokines in PDAC formation and have proved to interact with p38 pathway. Consistent with other studies [35–37], our Western blot analysis indicated that FGF-2, IGF-1 and EGF significantly induced p38 phosphorylation in PDAC cells (Fig. 5a). Next, we investigated whether these cytokines took part in LRG-1 mediated p38 phosphorylation, and found that LRG-1 induced upregulation of p38 phosphorylation was almost completely abolished by the EGFR inhibitor Erlotinib, while the FGFR and IGFR-1 inhibitors had little effect on LRG-1 induced upregulation of p38 phosphorylation (Fig. 5b).

### EGFR is critical for LRG-1-mediated malignant behaviors of PDAC cells

Recently, researchers found that LRG-1 could bind directly to endoglin, promoting the pro-angiogenic Smad1/5 signaling pathway (PMID: 23868260). We were therefore curious to know whether LRG-1 bound directly to the EGFR to enhance its activation and further induce p38 phosphorylation. Therefore, we performed Co-immunoprecipitation (Co-IP) assays to determine whether LRG-1 might bind directly to EGFR. Co-IP assay showed that EGFR and LRG-1 could form a complex, but no obvious interaction between FGFR and IGF-1R with LRG-1 was observed (Fig. 5c). We further tested the expression of EGFR and p-p38 in both PDAC tissues and normal pancreas tissues and found the expression of EGFR and p-p38 was significantly higher in PDAC tissues than that in normal pancreas tissues (Fig. 5d). The expressions of LRG-1, EGFR, p-p38 were evaluated in all PDAC tissues, the correlation analysis showed that EGFR (r = 0.224, *P* = 0.012) and p-p38 (r = 0.507, *P* < 0.001) expression was positively correlated with LRG-1 expression.

**Fig. 6:**
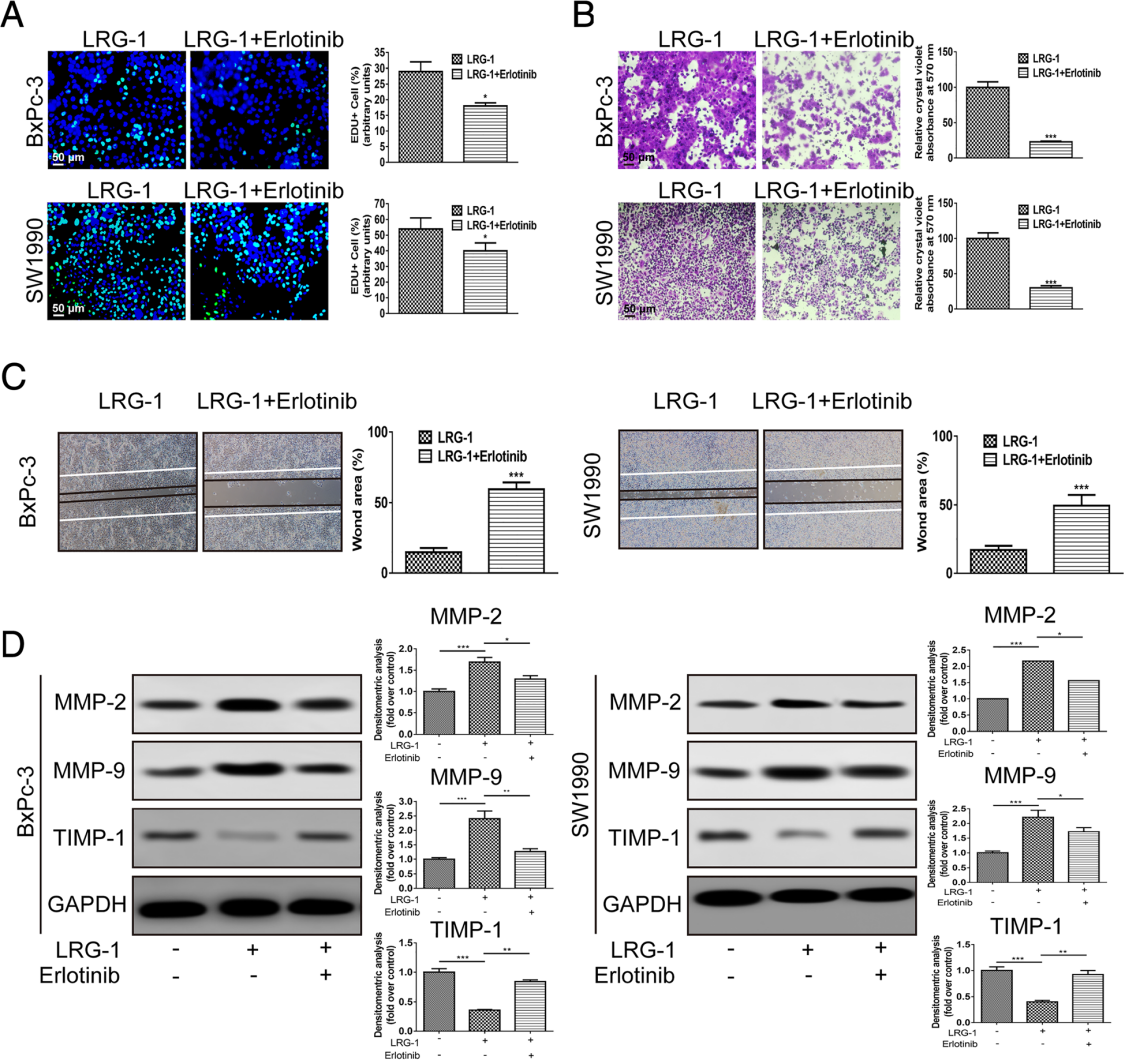
EGFR is critical for LRG-1-mediated malignant behavior of PDAC cells. **a** EdU incorporation assay, (**b**) Transwell assay and (**c**) Wound-healing assay in PDAC cells after 48 h incubation with LRG-1 (500 ng/ml) or LRG-1 + erlotinib (5 μM). **d** The protein level of MMP-2, MMP-9 and TIMP-1 in PDAC cells after 3-day incubation with LRG-1 (500 ng/ml) or LRG-1 + erlotinib (5 μM). Data are presented as the mean ± SD, *p < 0.05; **p < 0.01; ***p < 0.001

To determine whether the LRG-1-induced malignant behavior of PDAC cells was mediated by EGFR. EdU, Transwell assays and wound healing assays were performed. The results demonstrated that the promoting effect induced by LRG-1 was restored when Erlotinib was added in the BxPc-3 cell and SW1990 cell cultures (Fig. 6a, b and c). Erlotinib also reversed the upregulation of the protein levels of MMP-2 and MMP-9 induced by LRG-1 (Fig. 6d). These results indicate that EGFR played a crucial role in LRG-1-mediated malignant behavior of PDAC cells.

## Discussion

The incidence of PDAC is increasing rapidly during the past five years, accounting for about 90% of all pancreatic malignancies [38]. Current therapeutic approaches for PDAC mainly include pancreatic surgery, cytotoxic medication and radiation therapy [39]. However, survival of PDAC patients remains unsatisfied. Although some molecular-targeting therapies have been developed in recent years, survival benefits are still very limited. The unfavorable outcomes of these approaches could be ascribed to the poor understanding about the mechanisms underlying the pathogenesis of PDAC, knowing that the key oncogene causing PDAC remains undiscovered. Thus, it is urgent to find the relevant signaling pathways or target proteins, and clarify the exact mechanism about how PDAC develops and progresses.

In the present study, we found that LRG-1, a kind of secretive glycoprotein, could serve as an efficient biomarker for predicting the prognosis of PDAC patients. LRG-1 has been proven to be associated with inflammation and autoimmune disease in the past few years [40, 41]. Shinzaki et al. demonstrated that LRG-1 was a serum biomarker of mucosal healing in ulcerative colitis (UC) and serum LRG-1 concentrations in active UC patients was significantly higher than that in patients who had complete mucosal healing and deep remission [40, 42]. In osteoarthritis, tumor necrosis factor-α (TNF-α) induced LRG-1 expression in the subchondral bone and articular cartilage, and LRG-1 contributed to angiogenesis-coupled de novo bone formation in the subchondral bone of osteoarthritis joints [27]. Persistent inflammation could be an overture for malignant transformation of the normal tissue. Besides LRG-1 overexpression of in patients with inflammatory disease, LRG-1 expression of was also significantly higher in patients with malignancy. LRG-1 expression (serum or immunohistochemical staining) in patients with gastric cancer was higher than that in healthy controls, and LRG-1 expression increased with the progression of the pathological stage [43]. What’s more, LRG-1 expression was high in the malignant tissues of patients with colorectal cancer [17] and endometrial carcinoma [18], and it was correlated with tumor stage and lymph node metastasis. In patients with non-small cell lung cancer, serum LRG-1 expression was significantly higher than that in healthy volunteers, and LRG-1 was found as an outstanding tool in predicting prognosis and relapse [19]. It was found that in our study that LRG-1 expression in PDAC tissues was significantly higher than that in adjacent normal tissues. Survival analysis suggested that LRG-1 was an independent prognostic factor, and subsequent regression analysis indicated that LRG-1 level was correlated with more advanced tumor stage, higher CA 125 and CA 19–9 levels.

The malignant behavior of PDAC cells, including over proliferation, invasion and migration, is the pathophysiological characteristics in the development and progression of PDAC. Here, we demonstrated that LRG-1 could promote the viability, proliferation, migration and invasion of PDAC cells. Our in vivo experiment also showed that the tumor weight and size in mice injected with LRG-1 over-expressing PDAC cells exhibited obviously increased than that in mice injected with control PDAC cells. On the contrary, the tumor burden in nude mice injected with LRG-1 knockdown cells was significantly lower than that in control mice. This appeared to be consistent with previous publications demonstrating a potential tumor-promoting effect of LRG-1 on other malignancies, such as hepatocellular carcinoma [44], glioblastoma [20, 45] and colon cancer [46].

Furthermore, we found that the positive action in carcinogenesis was owing to the enhancing effect of LRG-1 on p38/MAPK signaling pathway in PDAC cells. MAPK signaling played important roles in regulating tumorigenesis, metastasis and chemoresistance of PDAC [47, 48]. Among all the downstream proteins of MAPK signaling tested in our experiments, the level of p38 phosphorylated was significantly increased after LRG-1 treatment, while the level of JNK and ERK phosphorylated remained unaffected. In addition, inhibition of the p38 pathway was sufficient to block LRG-1-induced malignant behavior of PDAC cells. This finding was also in line with a previous study reporting that LRG-1 significantly induced p38 phosphorylation in human bone marrow mesenchymal stem cells, thus promoting their migration and aberrant bone formation [27].

EGFR is a transmembrane glycoprotein that is conserved and overexpressed in pancreatic cancer [49]. EGFR over-expression has been confirmed to confer a poor survival, correlating with a more advanced stage and the presence of metastasis in pancreatic cancer [50]. EGFR phosphorylation initiates downstream signaling cascades such as MAPK, PI3K/Akt and Src pathways, which have been implicated in tumorigenesis by affecting cell proliferation, invasion and metastasis [51]. Interestingly, we found that EGFR inhibitor reduced LRG-1-induced p38 phosphorylation remarkably. Consistently, EdU incorporation assay and transwell assays showed that LRG-1 induced cell proliferation and invasion were almost abolished by the EGFR inhibitor. Further Co-IP assay revealed that LRG-1 could bind directly to EGFR to form a complex. Similarly, a previous study found that LRG-1 could bind to endoglin, a TGF-β accessory receptor, promoting the pro-angiogenic Smad1/5 signaling pathway [15].

## Conclusion

The expression of LRG-1 in PDAC tissue was significantly higher than that in adjacent normal tissue, and high LRG-1 expression predicted poor survival and aan dvanced tumor stage. LRG-1 remarkably promoted the viability, proliferation, migration and invasion of PDAC cells and facilitated tumor growth in vivo. This kind of bioactivity of LRG-1 may be ascribed to its selective interaction with EGFR and subsequent activation of the p38/MAPK signaling pathway. LRG-1 could serve as a promising biomarker for predicting prognosis in PDAC patients. Targeting LRG-1 or its downstream pathway may provide a novel and efficient strategy for the treatment of PDAC.

## Abbreviations

CA: Carbohydrate antigen
Co-IP: Co-immunoprecipitation
EGFR: Epidermal growth factor receptor
EMT: Epithelial-mesenchymal transition
FGF: Fibroblast growth factor
IGF-1: Insulin-like growth factor1
IHC: Immunohistochemistry
LRG-1: Leucine-rich-alpha-2-glycoprotein-1
MAPK: Mitogen-activated protein kinase
MMP: Matrix metalloproteinase
PDAC: Pancreatic ductal adenocarcinoma
SD: Standard deviation
SPF: Specific pathogen-free
TGF-β: Transforming growth factor β
TIMP-1: Tissue inhibitors of metalloproteinase-1
